# Antimicrobial data set and occurrence of acute kidney injury in patients admitted to a hospital in Western Pará, Brazil

**DOI:** 10.1016/j.dib.2025.111498

**Published:** 2025-03-24

**Authors:** Hiago Sousa Pinheiro, Camila Castilho Moraes, Géssica Aleane Moraes Esquerdo, Elenn Suzany Pereira Aranha, Luige Pinho Moraes, Tânia Mara Pires Moraes, Waldiney Pires Moraes

**Affiliations:** a Postgraduate Program in Biosciences at the Federal University of Western Pará, Brazil; b Postgraduate Program in Society, Nature and Development at the Federal University of West Pará, Brazil; c Postgraduate Program in Health Sciences at the Federal University of Western Pará, Brazil; d State University of Pará, Brazil; e Institute of Collective Health of the Federal University of Western Pará, Brazil

**Keywords:** Antibiotic therapy, Adverse drug reaction, Nephrotoxicity and acute kidney injury

## Abstract

In hospital units, the evaluation and pharmaceutical follow-up of medical prescriptions is an important source for pharmaceutical care and pharmaceutical clinical services. One common problem that has high hospital incidence rates is the occurrence of acute kidney injury (AKI). Pharmacovigilance among other activities is implemented in hospitals for the purpose of receiving and monitoring reports of adverse effects related to medications administered to patients. The survey evaluated the incidence of acute kidney injury in patients hospitalized and exposed to antimicrobials in a public hospital in the state of Pará, Brazil. A prospective and observational cohort was carried out, whose outcome of interest is the occurrence of AKI in patients admitted to the hospital between October 2018 and January 2019. The data were recorded and stored in a database, then descriptive analysis was performed in the GraphPad Prism 6.0 program. Quantitative variables were expressed as standard deviation (SD) of the mean and the number of cases as a percentage. We collected data from 70 patients who were admitted to the hospital and needed to use any of the antimicrobials selected in the observation period during hospital treatment. The survey results showed that mostly male (64.29%; *n =* 45). Age ranged from 19 to 96 years, with a mean of 52.49 years (SD = 20.31). The patients included were mostly from the oncology clinic (34.29%; *n =* 24) those that had had surgery (27.14%; *n =* 19). Most critically ill patients admitted to the adult ICU (26.47 %; *n =* 9) developed AKI. Regarding the number of medications used by patients, there was a variation from 5 to 17, with a mean of 10.26 (SD = 2.90) medications prescribed per patient. In the data regarding the antimicrobials, most patients took ceftriaxone (*n =* 29), cefepime (*n =* 27) and piperacillin/tazobactam (*n =* 23). In terms of the number of antimicrobials prescribed per patient, 60% (*n =* 42) of patients took only one, 30% (*n =* 21) took two and 10% (*n =* 7) took three or more antimicrobials for treatment of infections. The plasma concentrations of vancomycin ranged from 3.0 µg/mL to 22.5 µg/mL. Of the 10 samples collected, 10.0% (*n =* 1) were above the therapeutic range established by the literature (between 10 to 20 µg/mL), 30.0% (*n =* 3) were within the reference values and 60.0% (*n =* 6) of the patients had values below the reference values. Patients who developed AKI (60.0%; *n =* 6) during vancomycin use had concentration values between 3 µg/mL and 15.9 µg/mL, most of whom had values below the recommended therapeutic range. Of these patients with AKI, 83.33% (*n =* 5) used more than one nephrotoxic antimicrobial during hospital treatment. The concentrations of patients who were not diagnosed with AKI (40.0%; *n =* 4) ranged from 3.0 µg/mL to 22.5 µg/mL.

Specifications Table

**Table:** 

Subject	Pharmacology, Antimicrobial
Specific subject area	Data sets of antimicrobials in hospitalized patients with acute kidney injury.
Data format	Raw, analyzed, filtered.
Type of data	Tables
Data collection	The study was a prospective and observational cohort in hospitalized patients, in the time interval from October 2018 to January 2019. Patients were evaluated according to inclusion and exclusion criteria on the fifth day of admission. The medical records, prescriptions and laboratory test results available in the patients' records were used as sources for data collection. The included participants were interviewed and followed up to identify the development of AKI according to KDIGO guidelines, and plasma quantification of vancomycin concentrations in patients who used this antibiotic was also performed.
Data source location	The study was developed at Hospital Regional do Baixo Amazonas (HRBA), located in the municipality of Santarém (Pará). This institution is a public health unit that is owned by the Government of Pará and is administered by the Instituto Social Mais Saúde. It currently has 153 beds.
Data accessibility	Repository name: Mendeley data Data identification number: 10.17632/s58z23mvpw.4 Direct URL to data: https://data.mendeley.com/datasets/s58z23mvpw/4

## 1 Value of the Data

• These data will aid future scientific research related to the use of antimicrobials and the occurrence of acute kidney injury. This research could contribute to medical knowledge and the development of best practices in the treatment of infections.
• These data can be used for comparison with similar data from other hospitals and regions and make it possible to obtain answers that may influence the occurrence of acute kidney injury.
• These data help us to understand the relationship between the development of acute kidney injury and antimicrobial use and there may be interest in monitoring the prevalence of acute kidney injury in a given region and its relationship with antimicrobial use. The compilation of the data can help us to evaluate and improve clinical practices linked to the use of antimicrobials.

## 2 Background

Bacterial infections have led to numerous hospital admissions around the world and antimicrobials are their main form of treatment [1]. The problem is when they are used indiscriminately or inappropriately since this can lead to complications, including acute kidney injury (AKI) [2].

This injury can become a serious condition and can increase levels of hospital mortality and morbidity to alarming rates [3]. Understanding the risk factors, including the use of antimicrobials, is essential for the effective management of AKI, thus improving public health outcomes [4].

The population of the western region of Pará has its particularities in relation to bacterial resistance and access to antimicrobials, as well as specific epidemiological and socioeconomic characteristics. Thus, a study with this theme can provide valuable results on the pattern of antimicrobial use and its relationship with AKI in the regional context [5].

Knowing the association between the use of antimicrobials and the occurrence of AKI can help health professionals to implement preventive and educational interventions aimed at reducing the risks of acute kidney injury in hospitalized patients [6].

## 3 Data Description

The study began with 251 hospitalized patients who were given any of the antimicrobials selected in the observation period during hospital treatment. Of these, 181 were excluded from the research since they did not meet the inclusion criteria, leaving a total of 70 patients. Patients excluded because of their period of antimicrobial use was less than five days due to hospital discharge, death or intervention of the Hospital Infection Control Commission (CCIH) (alteration or suspended treatment).

The results show that the majority of the patients followed up in the study were male (64.29%; *n =* 45). Age ranged from 19 to 96 years, with a mean of 52.49 years (SD = 20.31). Regarding skin color, 88.57% (*n =* 62) of the patients self-reported as non-black. Regarding the BMI classification, 54.29% (*n =* 38) of the patients were considered as having a normal BMI. The mean age (55.32) of patients with AKI was higher than that of those who did not have this condition. Most patients (35.29%) who were classified as pre-obese developed AKI. The demographic characterization of the above population is described in Table 1.

**Table 1:** Demographic characterization and development of acute kidney injury in patients hospitalized and treated with nephrotoxic antimicrobials in a public hospital in western Pará.

Demographic data	Total (*n =* 70)	AKI (*n =* 34)	No AKI (*n =* 36)
	mean ± SD	mean ± SD	mean ± SD
**Age (years):**	52.49 ± 20.31	55.32 ± 20.06	49.81 ± 20.47
**Sex**	**% (n)**	**% (n)**	**% (n)**
Male	64.29 (45)	64.71 (22)	63.89 (23)
Female	35.71 (25)	35.29 (12)	36.11 (13)
**Skin color**	**% (n)**	**% (n)**	**% (n)**
Non-black	88.57 (62)	91.18 (31)	86.11 (31)
Black	11.43 (8)	8.82 (3)	13.89 (5)
**BMI**	**% (n)**	**% (n)**	**% (n)**
Underweight	12.86 (9)	5.88 (2)	19.44 (7)
Normal weight	54.29 (38)	50.00 (17)	58.33 (21)
Pre-obese	22.86 (16)	35.29 (12)	11.11 (4)
Obese I	7.14 (5)	5.88 (2)	8.33 (3)
Obese II	0.00 (0)	0.00 (0)	0.00 (0)
Obesity III	2.86 (2)	2.94 (1)	2.78 (1)

AKI: acute kidney Injury; SD: standard deviation; BMI: body mass index.

The patients included were mostly from the oncology clinic (34.29%; *n =* 24) and the surgical clinic (27.14%; *n =* 19). Most critically ill patients admitted to the adult ICU (26.47 %; *n =* 9) developed AKI. Among the health conditions identified as the cause of hospital admission, the most frequent were neoplasms (50%) or injuries and other consequences of external causes (17.14%).

According to the recording of the serum Cr dosage, an estimate of the glomerular filtration rate (GFR) was calculated at the beginning of the treatment for infection and a mean GFR of 101.4 mL/min/1.73m^2^ (SD = 33.9) was observed in the patients followed up. In patients who developed AKI, the GFR was observed at the beginning of treatment with a mean of 111.4 mL/min/1.73m^2^ (SD = 33.4) and a mean of 90.8 mL/min/1.73m^2^ (SD = 31.7) for those who did not develop AKI.

The presence of comorbidities on admission was described for 31.43% (*n =* 22) of the patients, and the most frequent were neoplasms (30.61%; *n =* 15), hypertension (30.61%; *n =* 15) and diabetes (24.49%; *n =* 12). Regarding the number of comorbidities, 25.71% (*n =* 18) of the patients had two and 5.71% (*n =* 4) had three or more comorbidities.

Regarding the characterization of the hospital care, sedation was used in 15.71% (*n =* 11) of the patients, chemotherapy treatment in 18.57% (*n =* 13), surgical procedures in 57.14% (*n =* 40) and mechanical ventilation in 17.14% (*n =* 12). All the patients who were followed up had some infection that justified the use of antimicrobials, and 17.14% (*n =* 12) had a diagnosis of sepsis. The length of stay in the hospital ranged from 6 to 86 days, with a mean of 26.5 days (SD = 18.39), with a total of 20% (*n =* 14) of the patients dying (Table 2). In terms of the evolution of the clinical picture of the patients who developed AKI, two underwent hemodialysis at some point during the use of the antimicrobials and death was observed for seven patients with AKI out of a total of 14 deaths.

**Table 2:** Clinical profile and development of acute kidney injury in patients hospitalized and treated with nephrotoxic antimicrobials in a public hospital in western Pará.

Clinical data	Total (*n =* 70)	AKI (*n =* 34)	No AKI (*n =* 36)
	Mean ± SD	Mean ± SD	Mean ± SD
**Length of hospital stay (days)**	26.5 ± 18.39	32.06 ± 21.09	21.25 ± 13.75
**GFR (mL/min/1.73 m^2^)**	**Mean ± SD**	**Mean ± SD**	**Mean ± SD**
At the beginning of the treatment for infection	101.4 ± 33.9	111.4 ± 33.4	90.8 ± 31.7
**Sector of origin**	**% (n)**	**% (n)**	**% (n)**
Adult ICU	17.14 (12)	26.47 (9)	8.33 (3)
Cancer clinic	34.29 (24)	29.41 (10)	38.89 (14)
General clinic	21.43 (15)	29.41 (10)	13.89 (5)
Surgical clinic	27.14 (19)	14.71 (5)	38.89 (14)
**Reason for admission**	**% (n)**	**% (n)**	**% (n)**
Infectious and parasitic diseases	1.43 (1)	2.94 (1)	0.00 (0)
Neoplasms	50.00 (35)	38.24 (13)	61.11 (22)
Diseases of the blood and hematopoietic organs	2.86 (2)	5.88 (2)	0.00 (0)
Diseases of the circulatory system	8.57 (6)	11.76 (4)	5.56 (2)
Diseases of the nervous system	1.43 (1)	0.00 (0)	2.78 (1)
Diseases of the eye and appendices	1.43 (1)	0.00 (0)	2.78 (1)
Diseases of the respiratory system	1.43 (1)	2.94 (1)	0.00 (0)
Diseases of the gastrointestinal tract	5 (7.14)	5.88 (2)	8.33 (3)
Diseases of the skin and subcutaneous tissue	1.43 (1)	2.94 (1)	0.00 (0)
Diseases of the musculoskeletal system	2.86 (2)	0.00 (0)	5.56 (2)
Diseases of the genitourinary system	1.43 (1)	0.00 (0)	2.78 (1)
Lesions or other consequences of external causes	17.14 (12)	23.53 (8)	11.11 (4)
Symptoms, signs and abnormal findings of clinical and laboratory exams	2.86 (2)	5.88 (2)	0.00 (0)
**Number of comorbidities**	**% (n)**	**% (n)**	**% (n)**
0	68.57 (48)	61.76 (21)	75.00 (27)
- 1 to 2	25.71 (18)	32.35 (11)	19.44 (7)
> 2	5.71 (4)	5.88 (2)	5.56 (2)
**Comorbidities**	**% (n)**	**% (n)**	**% (n)**
Diabetes	24.49 (12)	24.14 (7)	25.00 (5)
High blood pressure	30.61 (15)	24.14 (7)	40.00 (8)
Heart disease	10.20 (5)	13.79 (4)	5.00 (1)
Neoplasms	30.61 (15)	31.03 (9)	30.00 (6)
Other	4.08 (2)	6.90 (2)	0.00 (0)
**Hospital treatment**	**% (n)**	**% (n)**	**% (n)**
Chemotherapy	18.57 (13)	14.71 (5)	22.22 (8)
Sedation	15.71 (11)	23.53 (8)	8.33 (3)
Mechanical ventilation	17.14 (12)	26.47 (9)	8.33 (3)
Surgical procedure	57.14 (40)	52.94 (18)	61.11 (22)
Sepsis	17.14 (12)	26.47 (9)	8.33 (3)
**Outcome**	**% (n)**	**% (n)**	**% (n)**
Discharge	80.00 (56)	79.41 (27)	80.56 (29)
Death	20.00 (14)	20.59 (7)	19.44 (7)

AKI: acute kidney injury; SD: standard deviation; GFR: glomerular filtration rate; ICU: intensive care unit.

Regarding the number of medications used by the patients, there was a variation from 5 to 17 medications, with a mean of 10.26 (SD = 2.90) prescribed per patient. Regarding antimicrobial data, most patients used ceftriaxone (*n =* 29), cefepime (*n =* 27) and piperacillin/tazobactam (*n =* 23). Regarding the number of antimicrobials prescribed per patient, 60% (*n =* 42) of patients used only one, 30% (*n =* 21) used two and 10% (*n =* 7) used three or more antimicrobials for treatment of the infection.

In relation to the patients who used amphotericin B in the treatment of infectious diseases, 100% (*n =* 2) had AKI with a mean serum Cr variation of 1.35 mg/dL (SD = 0.77) and 60% (*n =* 6) of patients who received the antibiotic vancomycin were diagnosed with AKI, with a mean serum Cr variation of 1.27 mg/dL (SD = 2.42) during antibiotic use (Table 3).

**Table 3:** Variation in serum creatinine in the development of acute kidney injury and antimicrobial use in patients of a public hospital in western Pará.

Antimicrobial	No. of patients	AKI		No AKI	
		% (n)	∆ serum Cr, mean ± SD	% (n)	∆ serum Cr, mean ± SD
Ceftriaxone	29	31.03 (9)	0.65 ± 0.48	68.97 (20)	-0.12 ± 0.29
Cefepime	27	37.04 (10)	0.55 ± 0.48	62.96 (17)	-0.15 ± 0.33
Piperacillin/tazobactam	23	47.83 (11)	1 ± 1.75	52.17 (12)	0.08 ± 0.24
Ciprofloxacin,	13	30.77 (4)	0.27 ± 0.05	69.23 (9)	0.2 ± 0.34
Vancomycin	For 10	60.00 (6)	1.27 ± 2.42	40.00 (4)	-0.05 ± 0.24
Amphotericin B	2	100.00 (2)	1.35 ± 0.77	0.00 (0)	0
Amikacin	2	50.00 (1)	0.3	50.00 (1)	-0.4
Gentamicin	1	0.00 (0)	0	100.00 (1)	-1

Source: Authors. AKI: acute kidney injury; Δ serum Cr (mg/dL): serum creatinine variation; SD: standard deviation.

Plasma concentrations of vancomycin collected in the period analyzed ranged from 3.0 µg/mL to 22.5 µg/mL. Of the 10 samples collected, 10.0% (*n =* 1) were greater than the therapeutic range established by the literature (between 10 to 20 µg/mL), 30.0% (*n =* 3) were within the reference values and 60.0% (*n =* 6) of the patients had values below the reference values. Patients who developed AKI (60.0%; *n =* 6) during vancomycin use had concentration values of between 3 µg/mL and 15.9 µg/mL, most of whom had values below the recommended therapeutic range. Of these patients with AKI, 83.33% (*n =* 5) used more than one nephrotoxic antimicrobial during hospital treatment. The vancomycin concentrations of patients who were not diagnosed with AKI (40.0%; *n =* 4) ranged from 3.0 µg/mL to 22.5 µg/mL.

## 4 Experimental Design, Materials and Methods

The study involved a prospective and observational cohort [7] whose outcome of interest is the occurrence of AKI in patients admitted to a public hospital in the city of Santarém, Pará state, Brazil, from October 2018 to January 2019, totaling 90 days of data collection.

The study included patients aged 18 years or over, who had no history or any record of kidney disease at the time of admission, who were treated with at least one of the antimicrobials with the potential for nephrotoxicity and had a treatment time of at least five days with these drugs and at least two laboratory tests occurring during treatment. The most commonly used antimicrobials were classified according to the treatment authorization spreadsheet of the hospital infection control commission (CCIH) and through the monthly consumption presented by the hospital management software (Tazy); these being amikacin, amphotericin B, cefepime, ceftriaxone, ciprofloxacin, gentamicin, piperacillin/tazobactam and vancomycin.

Patients under the age of 18 years, with antimicrobial treatment of less than five days, those who performed less than two laboratory tests during treatment and those with a history or any record of kidney disease, submitted or not to renal replacement therapy, were excluded from the study.

The incidence of AKI cases was determined by applying the guidelines of Kidney Disease: Improving Global Outcomes (KDIGO) [8], where*: SCr* ≥ 1.5 times higher than baseline, known or presumed to have occurred in one week and/or *SCr* ≥ 0.3 mg/dL (26 µmol/L) in a period of 48 h.

For cases that met the diagnostic criteria, the following criteria were applied from the KDIGO groups: when a variation in *SCr* occurred and was equal to or greater than 1.5x baseline *SCr*, but less than 2x baseline *SCr*, known to have or presumed to have occurred in the last 7 days and/or *SCr* ≥ 0.3 mg/dL (26 µmol/L) in a period of 48 h, this corresponds to stage I; a variation in SCr equal to or greater than 2x baseline *SCr*, but less than 3x baseline *SCr*, this corresponds to stage II; a variation in *SCr* equal to or greater than 3x baseline *SCr* or an increase in *SCr* to ≥ 4.0 mg/dL (354 µmol/L) or initiation of renal replacement therapy, this corresponds to stage III.

The data were recorded and stored in a database, and the analysis was descriptive and performed using the GraphPad Prism 6.0 program. The quantitative variables were expressed as the mean (SD) and as a percentage of the number of cases (%).

## Limitations

The limitations of the work lie in the unavailability of comprehensive longitudinal data on the health history of hospitalized patients, which could affect the complete analysis of the relationship between the administration of antimicrobials and the incidence of acute kidney injury.

## Ethics Statement

Considering the terms of resolution 466/2012 of the National Health Council, all the ethical requirements were met regarding studies involving human beings. The project was approved by the Board of Teaching and Research of the Regional Hospital of Baixo Amazonas (DEP/HRBA) and was submitted to and approved by the Research Ethics Committee of the Instituto Esperança de Ensino Superior (CEP/IESPES) under Opinion No. 2,902,838 on September 19, 2018 (CAAE – 91160418.8.0000.8070). All research participants signed the ICF and, in case of impossibility of decision on the part of the patient, this was signed by their legal guardian.

## CRediT Author Statement

**Hiago Sousa Pinheiro:** Conceptualization, methodology, programs, validation, formal analysis, research, data curation, writing. **Camila Castilho Moraes:** Conceptualization, methodology, validation, formal analysis, writing, essay. **Géssica Aleane Moraes Esquerdo:** Conceptualization, methodology, validation, formal analysis, writing. **Elenn Suzany Pereira Aranha:** Conceptualization, methodology, validation, formal analysis, writing. **Luige Pinho Moraes:** Methodology, validation, formal analysis, writing. **Tânia Mara Pires Moraes:** Conceptualization, methodology, data curation, writing, supervision, project administration. **Waldiney Pires Moraes:** Conceptualization, methodology, data curation, writing, supervision, project administration.

## Data Availability

Mendeley DataRESULTADO DE DADOS ANTIMICROBIANO E OCORRÊNCIA DE LESÃO RENAL AGUDA EM PACIENTES INTERNADOS EM UM HOSPITAL NO OESTE DO PARÁ, BRASIL. (Original data).
